# Critical Appraisal of Guidelines for Antithrombotic Therapy in Atrial Fibrillation Post-Percutaneous Coronary Intervention

**DOI:** 10.5334/gh.1104

**Published:** 2022-02-23

**Authors:** Yongqiang Fan, Gaoxing Zhang, Zhengzhipeng Zhang, Shaozhao Zhang, Menghui Liu, Yifen Lin, Yiquan Huang, Xiangbin Zhong, Xiaodong Zhuang, Xinxue Liao

**Affiliations:** 1 Department of Cardiology, The Jiangmen Central Hospital, Jiangmen, CN; 2 Department of Cardiology, The First first affiliated hospital of Sun Yat-Sen University, Guangzhou, CN; 3 NHC Key Laboratory of Assisted Circulation (Sun Yat-sen University), Guangzhou, People’s Republic of China, CN

**Keywords:** AGREE II, antithrombotic therapy, atrial fibrillation, percutaneous coronary intervention, guideline

## Abstract

**Objective:**

In our present study, our objective was to appraise guidelines on antithrombotic therapy in atrial fibrillation post-percutaneous coronary intervention and to explore the differences in treatment practices for better informed decision-making.

**Methods:**

We searched for English language guidelines published between January 2000 and December 2020 at MEDLINE, Embase and websites of guideline organizations. Guidelines with recommendations on antithrombotic regimens for patients with AF undergoing PCI were included. Appraisal of Guidelines for Research and Evaluation II (AGREE II) instrument was applied to assess guidelines. The reporting of conflicts of interest (COI) was evaluated separately by the RIGHT (Reporting Item for Practice Guidelines in Healthcare) checklist as supplementary items.

**Results:**

Sixteen guidelines were included, among which 13 (81.25%) were considered as ‘recommended’ and 1 (6.25%) as ‘unrecommended.’ The average scores of guidelines ranged from 55% to 88% (<60% as low quality, 60–70% as sufficient quality, and >70% as good quality). Among the 6 domains of AGREE II, scope and purpose (84%) and editorial independence(87%) were considered to be the fields in which CPGs performed best, evidenced by the highest mean AGREE II scores. The domains in which the reviewed CPGs received the lowest mean scores were stakeholder involvement (63%) and applicability (58%). The intraclass correlation coefficient scores were excellent in each domain. The overall quality of the selected CPGs was optimal, with the highest score in domain ‘scope and purpose’, and the lowest score in the domain ‘applicability.’ The reporting of COI was satisfactory.

**Conclusions:**

For the recommendations on antithrombotic strategies, guidelines with high AGREE II scores still exist discrepancy on the timing and selection. Current guidance documents on the treatment vary in methodological rigor and recommendations are not always consistent.

## Introduction

Atrial fibrillation (AF) affects roughly 33 million patients worldwide, about 30% of whom are complicated with coronary artery disease (CAD), and 5–10% of whom will undergo percutaneous coronary intervention (PCI) during their life [1,2]. Oral anticoagulation has been used as the first choice to prevent stroke and systemic embolism in patients with AF but has not been proved to avert stent thrombosis [3]. Dual antiplatelet therapy is confirmed to reduce the incidence of recurrent ischemic events and stent thrombosis but is less effective in reducing the impact of cardioembolic stroke associated with atrial fibrillation [4]. When AF patients encounter PCI, the relationship between anticoagulation and antiplatelet treatment should be balanced. However, the combination of antithrombotic agents, particularly triple antithrombotic therapy (TAT) with oral anticoagulation and dual antiplatelet therapy, will increase the risk of bleeding [5]. Thus choosing antithrombotic therapy for patients with atrial fibrillation who have undergone PCI is challenging. Clinical practice guidelines are systematically developed statements to help the practitioner to make rational decisions in specific clinical circumstances. There were more than 10 antithrombosis-relevant clinical practice guidelines (CPGs) published over the past two decades. Although most of them were claimed to be based on high-quality evidence, we found considerable variation in their recommendations, which may confuse clinicians. As the quality of these guidelines was unclear, critical appraisal of these guidelines is crucial. Therefore, we examined the availability, consistency and quality of CPGs for individuals who underwent PCI and AF. Our systematic review aimed to summarise recommendations and appraise the quality of internationally available antithrombosis solution CPGs.

## Methods

### Data sources and searches

**Figure 1:**
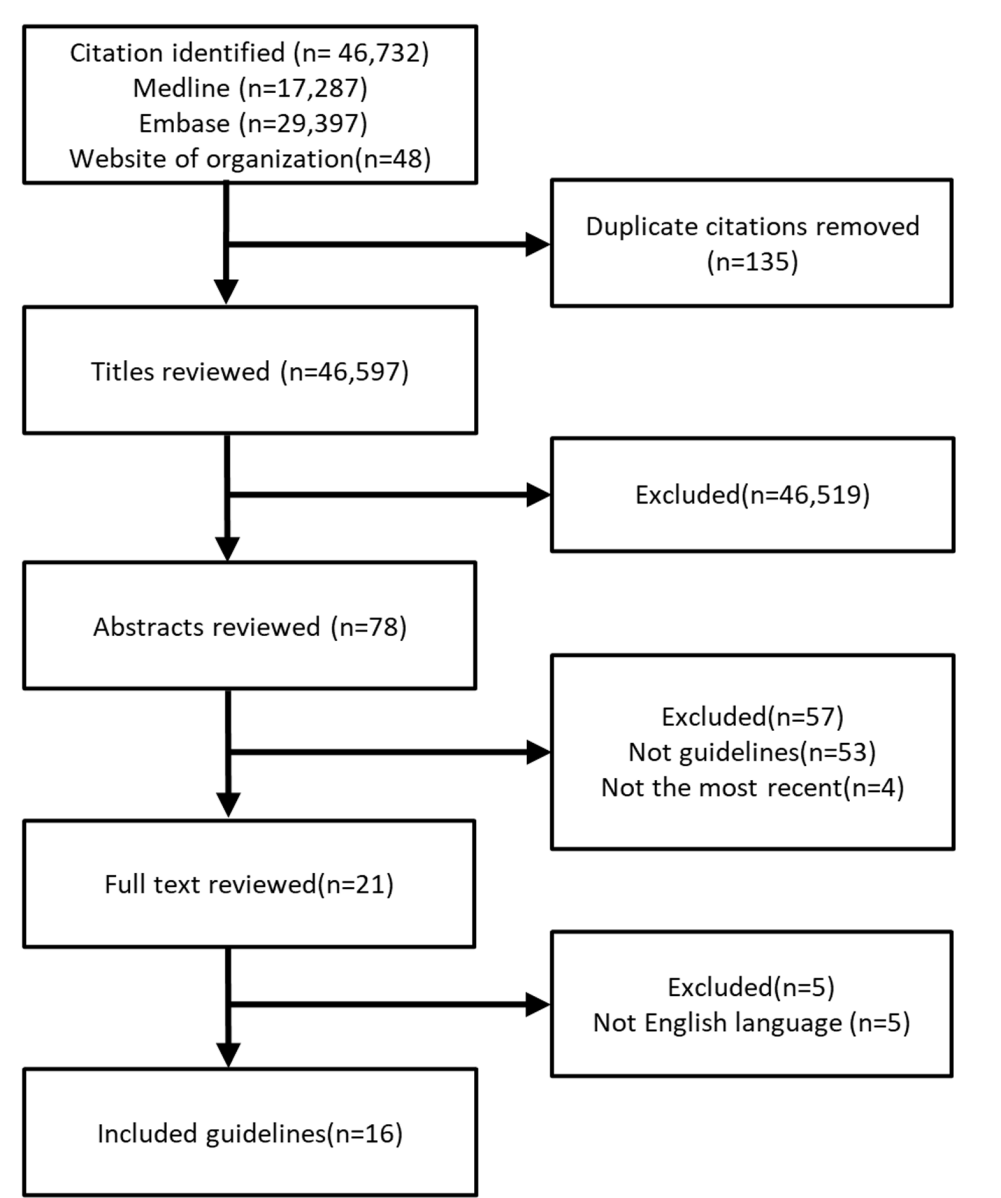
Flow diagram of inclusion/exclusion processes for the guidelines.

To identify appropriate guidelines, we systematically searched PubMed and EMBASE from 1 January 2000 to 1 December 2020, using keywords of ‘Percutaneous Coronary Intervention,’ ‘Acute Coronary Syndrome,’ ‘Myocardial Ischemia,’ ‘Antithrombotics,’ ‘Atrial Fibrillation,’ ‘Antiplatelet,’ and ‘guideline*.’ The National Guidelines Clearinghouse, a guideline-specific database, and ECRI Guidelines Trust were also searched as supplementary sources. Supplementary guidelines were available by searching websites of guideline organizations (see ***Figure 1***) Criteria for selection were as follows: (a) follow the definition of CPGs [6]: systematically develop statements to assist physicians and patients in determining appropriate medical care for specific clinical situations; (b) target groups included patients with AF and PCI;(c) contain recommendations on antithrombotic therapy for target patients; (d) are published in English. Two reviewers (F.Y.Q and Z.Z.Z.P) reviewed titles and abstracts independently and removed any inappropriate articles. The discrepancy was discussed and resolved by face-to-face discussion, or in case of persistent disagreement, by consultation with a third researcher. The final selection of articles was reviewed by the third reviewer (Z.S.Z).

### Quality Appraisal

Two independent cardiologists (F.Y.Q and Z.Z.Z.P), who were blinded to each other‘s ratings, assessed CPG quality using the Appraisal of Guidelines for Research and Evaluation Instrument, version II (AGREE-II). After that, one methodological expert (Z.X.D) evaluated CPGs in a blinded fashion. The AGREE tool was created by an international group of experts to support the process of development, assessment and reporting of CPGs [7,8]. Later, AGREE-II is a modification of the original AGREE to create a more comprehensive assessment of guideline [9]. The tool consists of 23 items in six domains:(a) scope and purpose; (b) stakeholder involvement; (c) rigor of development; (d) clarity of presentation; (e) applicability; (f) editorial independence. Each item scores from 1(strongly disagree) to 7(strongly agree). The final rating for each guideline is based on the average score for all domains. AGREE manual does not set a threshold, and reviewers and assessors should analyze the scores and take decisions according to their contexts and preferences. However, some authors have used some scores to determine this quality [10,11], although there is no evidence to define which threshold is better. Before each AGREE II domain assessment, a meeting was held to discuss the appraisal criteria according to the AGREE II manual and training tools.

Furthermore, the reporting of conflicts of interest (COI) was assessed in the present study. Not only in regard to the two items in domain 6 of AGREE II, but also four items from the RIGHT checklist [12] were appraised by one reviewer (F.Y.Q) and checked by a second reviewer (Z.Z.Z.P). Besides, we counted the proportion of guideline panel member-industry relationships and listed table detailing the main funder of each guideline (see table S1). Discrepancies were resolved by discussion.

### Data extraction and analysis

All recommendations about antithrombotic therapy in AF undergoing PCI from each included guideline were extracted by one reviewer (F.Y.Q). A second reviewer (Z.Z.Z.P) checked the completeness and accuracy of the results. A comparison of the recommendations has been shown in ***Table 2***. Each proposal was categorized into risk evaluation, drug selection and duration. All data relevant to the study are included in the article or uploaded as supplementary information. Agreement among reviewers was measured by intraclass correlation coefficient (ICC) calculated by SPSS version 19.0.

## Results

### Selected guidelines

The flow chart (***Figure 1***) shows the process of screening and selecting guidelines. Ultimately, a total of 16 guidelines met the inclusion criteria. The characteristics of the eligible guidelines are summarized in ***Table 1***, with COI and the average AGREE II scores. Four guidelines were from the European continent [13,14,15,16], with one from the United Kingdom [17], four from the United States [18,19,20,21], two from Canada [22,23], two from Australia and New Zealand [24,25], one from Japan [26], and two from Taiwan [27,28].

**Table 1:** Characteristics of 16 Guidelines on Antithrombotic Therapy in post-PCI Patients with AF.


GUIDELINES IDENTIFIER, YEAR	ORGANIZATION(S)	REGION	TARGET POPULATION	AGREE SCORE, %	CONFLICTS OF INTEREST	PROPORTION OF PANE MEMBERS WITH REPORTED INDUSTRY RELATIONSHIP	GUIDELINE STATUS

AHA/ACC, 2019	American Heart Association/American College of Cardiology	United Stated	AF	77	*SCI, *SCIR, DIR, DSFS, DTCO, DEMC, DADI	7/15	Strongly recommended

AHA/ACC, 2014	American Heart Association/American College of Cardiology	United Stated	NSTE-ACS	78	*SCI, *SCIR, DIR, DSFS, DTCO, DEMC, DADI	7/17	Strongly recommended

ACCF/AHA, 2013	American Heart Association/American College of Cardiology Foundation	United Stated	STE-ACS	76	*SCI, *SCIR, DIR, DSFS, DTCO, DEMC, DADI	12/23	Strongly recommended

Chest, 2018	American College of Chest	American	AF	78	*SCI, *SCIR, DIR, DSFS, DTCO, DEMC, DADI	8/12	Strongly recommended

CCS, 2018	Canadian Cardiovascular Society	Canada	CAD	76	*SCI, *SCIR, DSFS, DTCO, DADI	13/22	Strongly recommended

CCS, 2018	Canadian Cardiovascular Society	Canada	AF	74	*SCI, *SCIR, DSFS, DTCO, DADI	22/25	Strongly recommended

ESC, 2020	European Society of Cardiology	Europe	AF	80	*SCI, *SCIR, DSFS, DTCO, DEMC, DADI	22/25	Strongly recommended

ESC, 2020	European Society of Cardiology	Europe	NSTE-ACS	78	*SCI, *SCIR, DSFS, DTCO, DEMC, DADI	24/26	Strongly recommended

ESC, 2019	European Society of Cardiology	Europe	CCS	74	*SCI, *SCIR, DSFS, DTCO, DEMC, DADI	22/25	Strongly recommended

ESC, 2017	European Society of Cardiology	Europe	CAD	75	*SCI, *SCIR, DSFS, DTCO, DEMC, DADI	13/18	Strongly recommended

NICE, 2013	National Institute for Health and Care Excellence	United Kingdom	STE-ACS	88	*SCI, DSFS, DTCO, DEMC, DADI	8/15	Strongly recommended

NHFA/CSANZ,2016	National Heart Foundation of Australia/Cardiac Society of Australia and New Zealand	Australia and New Zealand	ACS	77	*SCI, DSFS, DTCO, DEMC, DAD	29/29	Strongly recommended

NHFA/CSANZ,2018	National Heart Foundation of Australia/Cardiac Society of Australia and New Zealand	Australia and New Zealand	AF	73	*SCI, DSFS, DTCO, DEMC, DAD	16/18	Strongly recommended

JCS, 2013	Japanese Circulation Society	Japan	AF	60	-	11/11	Recommended

TSC, 2016	Taiwan Society of Cardiology	Taiwan	AF	55	DIR, DSFS	3/27	Not recommended

TSC, 2018	Taiwan Society of Cardiology	Taiwan	NSTEMI	63	DIR, DSFS	-	Recommended


* Relationship with industry reported by at least 1 person. SCI = statement about conflicts of interest of panel members present; SCIR = statement about conflicts of interest of external peer reviews present; DIR = disclosure of the identities of peer reviews; DSFS = disclosure of the specific sources of funding for all stages of guideline development; DTCO = disclosure the types of COI (financial and nonfinancial) that are relevant to the guidelines; DEMC = disclosure of the evaluation and management of the COI; DADI = disclosure of how to access the declarations of interests; CAD = coronary artery disease; STE-ACS = ST-Elevation Acute Coronary Syndromes; NSTE-ACS = Non-ST-Elevation Acute Coronary Syndromes; SCAD = Stable Coronary Artery Disease; ACS = Acute Coronary Syndromes; AF = Atrial Fibrillation.

**Table 2:** Recommendations in Guidelines on Antithrombotic Therapy after PCI in Atrial Fibrillation.


GUIDELINE	RISK EVALUATION	ACS		ELECTIVE PCI/CCS		MONOTHERAPY
	
		TRIPLE THERAPY	DUAL THERAPY	TRIPLE THERAPY	DUAL THERAPY	

AHA2019 AF	CHA2DS2-VASc≥2	O+A+P for 4–6w(b)	P+V/N has lower risk of bleeding	/	/	/

AHA2014 NSTEMI	/	V+A+P should be minimized to the extent()	/	/	/	/

AHA2013 STEMI	CHA2DS2-VASc≥2	V+A+P should be minimized to the extent()	/	/	/	/

CHEST2018 AF	HAS-BLED (0–2)	O+A+P for 6 mo. (weak)	P+O up to 12 mo.	O+A+P for 1 mo. (weak)	P+O up to 12 mo.	O

	HAS-BLED ≥ 3	O+A+P for 1–3 mo. (weak)	P+O up to 12 mo.	O+A+P for 1 mo. (weak)	P+O up to 12 mo.	O

	HAS-BLED > CHA2DS2-VASc	/	P+O for 6–9 mo. (weak)	/	P+O for 6 mo. (weak)	O

CCS2018 Antiplatelet	Age < 65 and CHADS2 = 0	/	A+P for 12 mo. (Strong)	/	A+P for 6–12 mo. (Strong)	A+/-P

	Age ≥ 65 or CHADS2 ≥ 1	O+A+P for 6 mo. (weak)	P+O up to 12 mo.	/	P+O for1–12 mo. a P+O for3–12 mo. b	O

CCS2018 AF	Age ≥ 65 or CHADS2 ≥ 1	O+A+P for 6 mo. (strong)	P+O up to 12 mo.	/	P+O for1–12 mo. a P+O for3–12 mo. b	O

ESC, 2020 AF	High ischaemic risk	1w<O+A+P <1 mo. (a)	P+O up to 12 mo. ()	O+A+P <1 mo. (a)	P+O up to 12 mo.	O

	Bleeding risk outweighs	O+A+P≤1 w. ()	P+O up to 12 mo. ()	O+A+P≤1 w. ()	P+O up to 6 mo.	O

ESC2020 NSTEMI	CHA2DS2-vasc≥1	O+A+P≤1 w. ()	P+O up to 12 mo.	/	/	O

	High ischaemic risk	1w<O+A+P <1 mo. (a)	P+O up to 12 mo.	/	/	

	High bleeding risk	O+A+P≤1 w.	P+O up to 6 mo.	/	/	O after 6 mo.

ESC2019 CCS	stent thrombosis low	/	/	O+A+P≤1 w. (a)	/	O

	high ischaemic risk	/	/	1mo.<O+A+P <6 mo. (a)	/	O

ESC2017 DAPT	high ischaemic risk	O+A+P for 6 mo.(a)	/	/	/	O

	High bleeding risk	/	P+O up to 12 mo. (a)	/	/	O

NICE2013 MI	/	P+V up to 12 mo.	/	/	/	O

NFHA2016ACS	HAS-BLED <3	O+A+P for 3–6 mo.	A/P+O up to 12 mo.	/	/	O

	HAS-BLED ≥ 3	O+A+P for 1 mo.	A/P+O up to 12 mo.	/	/	O

	CHA2DS2-VASc = 1	/	A+P up to 12 mo.	/	/	O

NFHA2018 AF	high ischaemic risk	O+A+P for 1–6 mo.	A/P+O up to 12 mo.	O+A+P for 1 mo.	A/P+O until 12 mo.	O

	High bleeding risk	O+A+P for 1 mo.	A/P+O up to 12 mo.	O+A+P < 1w	A/P+O until 12 mo.	O

JCS2013 AF	/	Triple therapy may be considered	/	/	/	/

TSC2018 NSTEMI	CHA2DS2-VASc≥2	O+A+P for 1–6 mo. (a)	/	/	/	O

	High bleeding risk	/	P+O for 12 mo.	/	/	O

	high ischaemic risk	O+A+P for 1–6 mo.	P+O > 12 mo.	/	/	O

TSC2016 AF	/	O+A+P for 3–6 mo.	P+O up to 12 mo.	O+A+P for 1 mo.	P+O up to 12 mo.	O

	High bleeding risk	/	P+O for 12 mo.	O+A+P < 1 mo.	P+O for 3–6 mo.	O


V = Vitamin K Antagonists; N = non-VKA oral anticoagulants; O = Oral anticoagulants; A = aspirin; P = P2Y12 inhibitor; a Bare metal stents; b drug eluting stent; ACS: acute coronary syndrome; CCS: chronic coronary syndrome; PCI: Percutaneous coronary intervention.

### Quality assessment of guidelines

**Figure 2:**
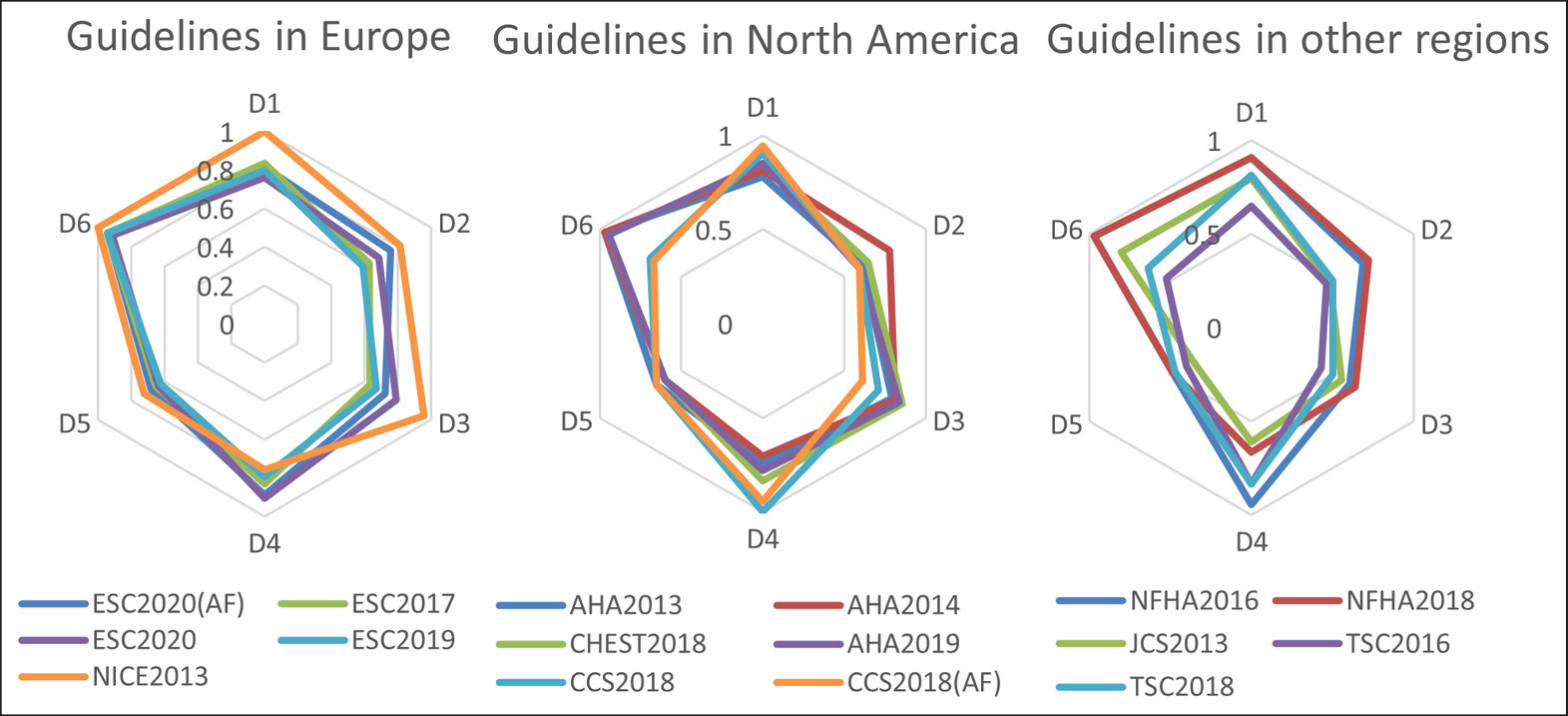
Rader charts of the AGREE II score distribution across 6 domains for the guidelines. ESC, European Society of Cardiology; NICE, National Institute for Health and Care Excellence; AHA, American Heart Association; CCS, Canadian Cardiovascular Society; JCS, Japanese Circulation Society; NHFA, National Heart Foundation of Australia; TSC, Taiwan Society of Cardiology; D1, Scope and Purpose; D2, Stakeholder Involvement; D3, Rigor of Development; D4, Clarity of Presentation; D5, Applicability; D6, Editorial Independence.

***Figure 2*** shows the final scores of each domain for the guidelines, which indicate the final score for each guideline in the six domains. The position closer to the periphery represents higher domain scores and vice versa. The average AGREE II scores of the guidelines ranged from 55% to 88%. The selected CPGs received the highest score in domain ‘scope and purpose,’ and the lowest score in the domain ‘editorial independence.’ Overall, ICC scores were >85% in each domain, suggesting considerable agreement between the reviewers. (see table S2).

#### Scope and purpose

Guidelines in this domain were graded (median, 84%; Range: 65 to 100%). The highest score in this domain was 100%, as the guidelines clearly define their scope and global objectives, as well as the relevant clinical areas and target populations [17].

#### Stakeholder involvement

For stakeholder involvement, the guidelines appraised got the second lowest scores (median, 63%; Range: 46 to 81%). Six guidelines (37.5%) scored lower than 60% for domain ‘stakeholder involvement [14,16,22,23,26,27].’ No guidelines involved patients or their representatives, taking the preferences of the target population in the formulation of the guidelines into account.

#### Rigor of development

This domain showed a relatively good score among the guidelines, with an range from 43 to 96%. Three guidelines (18.75%) scored lower than 60% [26,27,28] because of the lack of systematic methods to report finding or evaluating evidence. Only four guidelines (NICE2013, AHA2013, AHA2014, AHA2019) described the process of making a final decision [17,18,19,21].

#### Clarity of presentation

For domain ‘clarity of presentation,’ most of the guidelines scored around 80, with the median score of 82% (IQR: 61 to 100%). No guideline scored less than 60%, as most relevant recommendations in all guidelines are easily found with the recommended level, such as SOR and LOE.

#### Applicability

Applicability was by far the domain with the lowest ratings (median 58%; IQR: 36 to 72%). In fact, this was the only domain that did not reach the cut-off score of 60, and in which the NICE2013 guidelines received higher scores (72). In general, information regarding potential cost implications, organizational barriers and tools for application can be hardly found in most guidelines. Furthermore, only guidelines from the European Society of Cardiology (ESC), Canadian Cardiovascular Society (CCS) and the American Heart Association (AHA) provided educational tools and implementation programmers to help clinicians to put recommendations into practice. Only the NICE guidance considers cost-effectiveness, involving health economists into the guidance group, including evidence on health economics, and discussing the budgetary implications behind the recommendations.

#### Editorial independence

The greatest range of scores was observed in this domain (median 87%; range: 53, 100%). All guidelines got high scores, excluding the guidelines from TSC, all of which indicated a score above 70%. Competing interests, including financial and intellectual, were poorly addressed in 13 of the included guidelines and yielded low scores. Although most of the guidelines disclosed their conflicts of interest (COI), the quality of disclosure was not ideal. They provide little information about any form of COI management in either tabular or narrative form. Only 2013 NICE has summarized the COI process for identifying, managing and reporting during the guideline development [17].

### Synthesis of recommendations

**Figure 3:**
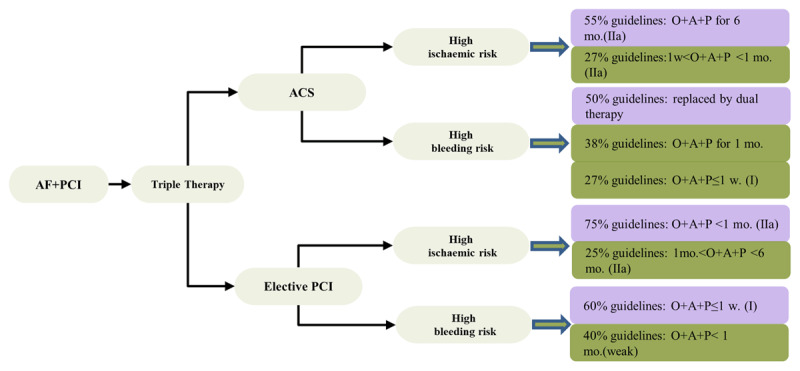
Flowcharts for the controversial clinical scenarios. O, Oral anticoagulation; A, Aspirin; P, P2Y12 inhibitor.

The included guidance documents addressed two significant themes: risk evaluation and triple antithrombotic therapy. ***Figure 3*** showed key recommendations and their inconsistencies.

#### Risk evaluation

The recommendation of six guidance documents (Chest 2018,CCS 2018,CCS 2018AF, ESC 2020, NFHA 2018, TSC 2016) was classified by ACS or stable heart disease, and only two guidelines [15,17] were not categorized systematically. The stroke risk score (CHA2DS2-VASc) was mentioned on nine guidelines [16,18,20,21,22,23,24,28], while bleeding score (HAS-BLED) was found in six guidelines [13,16,22,26,29,30]. In the meantime, five guidelines (CHEST 2018, ESC 2020, NHFA 2016, TSC 2016, TSC 2018) provided recommendations on both ischemic and bleeding scores. Three guidelines gave a treatment plan according to the type of stent, an essential factor in risk assessment, from CCS 2018, CCS2018(AF), TSC 2016.

#### Treatment

TAT, as the first choice from a majority of guidelines, was considered for ACS by all but three documents (CCS 2018, NICE 2013, JCS 2013). The duration of TAT is generally concentrated between 1 and 6 months, and is divided into different periods according to ischemic risk and hemorrhagenic risk. As for patients with ACS, six of the 11 guidelines (55%) suggested triple therapy for 6 months while four of 8 guidelines (50%) supported dual therapy instead of TAT in high bleeding risk (***Figure 3***). Only two guidelines [24,27] suggested TAT for 3–6 months, from 2016 NHFA and 2016 TSC. Three guidelines [21,25,27] indicated that the addition of ticagrelor or prasugrel to triple therapy is not recommended because of the lack of evidence of bleeding associated with OAC. No DAT is involved in three guidelines, such as AHA2014, AHA2013, ESC2015, which were published before the study of PIONEER. Using OAC alone was suggested to treat for a long time, following dual therapy.

## Discussion

In summary, 16 guidelines were identified. It was the first systematic review of the international antithrombosis guidelines. Using the AGREE II appraisal instrument to assess guidelines, we found the overall quality of these guidelines to be relatively high, though deficiencies still existed. Thirteen CPGs were considered to be high quality and suitable for recommendation to clinical practitioners and policymakers. The guidelines issued in Europe, in particular, were of high methodological and reporting quality [16,17]. Although one CPG used the GRADE system to classify the quality of evidence, its final scores of AGREE II were <60%, indicating that the methodological quality was insufficient [27]. Guidance documents assessed in our study performed well in the domains of scope and purpose (domain 1) and clarity of presentation (domain 4), but poorly in the domain of applicability (domain 5).

The AGREE II tool was often used to evaluate the quality of clinical guidelines in different specialties [29]. Previous studies mostly have shown the low scoring domains in domain 2 ‘stakeholder involvement,’ domain 3 ‘rigor of development,’ and domain 5 ‘applicability,’ reflecting the common problem of the guidelines in different disciplines. In present study, it is somewhat reassuring to note that most of these antithrombotic guidelines, especially from ESC, AHA/ACC, and NICE, got relatively good scores in the domain ‘rigor of development.’ This suggests that most guidelines are developed in strict accordance with evidence-based principles. However, two domains, ‘stakeholder involvement’ and ‘applicability,’ received a low score like other studies. At the same time, the lack of the views and preferences from the target population will limit the application of the guideline in various scenarios. Improving this item would help the wider audience get a better understanding of clinical guidelines. Also, the information of the individuals of the development group was not unveiled well in many guidelines. The incomplete professional groups may lead to the lower scores in the ‘applicability’ domain. In this domain, few guidelines pay attention to the facilitators or barriers to its application. For example, VKA was widely accepted in patients with AF, but its non-adherence was significantly higher compared with NOAC users [30]. In view of this phenomenon, recommendations involving management in community health services might help their adherence.

The Conflicts of Interest (COI) reports have become an integral part of the development of guidelines, which help optimize the trustworthiness of guidelines by controlling the risk of bias associated with COI and improving guideline credibility [12]. In selected guidelines, COI may influence the recommendations for the selection and duration of TAT. Except for 2018 TSC and 2013 JCS, all the guidelines got good scores in this domain. However, the items were still not comprehensive enough [12]. We systematically evaluated these items and found that the majority of these guidelines get good scores except for: ‘the role of the funder(s) in guideline development, dissemination, and implementation.’ The supplement of this item would significantly increase the credibility of the guidelines. As mentioned above, 2018 TSC and 2013 JCS provided little information about COI. Regrettably, most guidelines fail to provide the relationship between the COI and recommendation in which they were written, except 2018 CCS. More attention should be paid to COI when specified in the guidelines, and authors with COI should avoid voting when necessary.

In recent years, although numerous CPGs have been issued, the quality of CPGs has been uneven. Due to the possibility of recommendations of poor quality CPGs delaying treatment, it is particularly important to identify and develop high-quality CPGs for clinicians and health care professionals to use. Policymakers should pay more attention on eliciting the opinions of target populations and declarations of interest in the next 10 years. As to the organization, it is essential to update regularly and expand awareness through apps or brochures. Our study demonstrated that guidelines with better methodological quality also had greater reporting quality. Therefore, using both the AGREE II tool and COI to assess the quality of CPGs, we were able to identify the possible gaps in the different aspects as well as areas for further improvement.

### Strengths and Limitations

In the present study, it was the first systematic review of the international antithrombosis guidelines when AF encounters PCI. In addition to using AGREE II instrument, we also used RIGHT to further evaluate COI.

Our study has certain limitations. First, we included only the CPGs written in English, which could have limited the diversity of regions of origin. In spite of this limitation, we had selected a wide range of CPGs produced in different areas of the world. Second, the numbers and specialty of reviewers are the deficiency of our study. However, the whole evaluation process was transparent and independent by each reviewer. Third, we can have a direct impression of guidelines through the AGREE II tool. However, there is no inevitability between the score and the reliability of the specific recommendation. Finally, we list but do not address discrepancies across guidelines.

## Conclusion

Our systematic appraisal of CPGs found that the overall quality of a large proportion of antithrombotic guidelines is optimal, though defects existed in ‘Applicability’ and ‘Stakeholder Involvement.’ Current guidance documents on the treatment vary in methodological rigor and recommendations are not always consistent. The clinical decisions should be made on a case-by-case basis.

## Additional Files

The additional files for this article can be found as follows:

10.5334/gh.1104.s1 Table S1. The top-five main funder of eligible guidelines.

10.5334/gh.1104.s2 Table S2. AGREE II domain and overall assessment for eligible guidelines.
