# Supplementary material for: Critical Appraisal of Guidelines for Antithrombotic Therapy in Atrial Fibrillation Post-Percutaneous Coronary Intervention
Source: Glob Heart. 2022 Feb 23;17(1):14. doi: 10.5334/gh.1104 (PMC8877875; doi:10.5334/gh.1104)
Supplement: Table S1. — The top-five main funder of eligible guidelines. [file gh-17-1-1104-s1.pdf]

table S1

Table S1 The top-five main funder of eligible guidelines.

Guidelines Identifier, Year	1st	2nd	3rd	4th	5th
**AHA/ACC, 2019**	Medtronic (3/15)	Pfizer (3/15)	Bayer (2/15)	BI (2/15)	Johnson & Johnson(2/15)
**AHA/ACC, 2014**	Abbott (3/17)	Astra Zeneca (3/17)	Amgen (3/17)	Merck (3/17)	Pfizer (2/17)
**ACCF/AHA, 2013**	Astra Zeneca (4/23)	Sanofi (4/23)	Medtronic (3/23)	Merck (3/23)	Novartis (3/23)
**Chest,2018**	BI (6/12)	Bayer (5/12)	Medtronic (3/12）	Pfizer (3/12)	Astra Zeneca (2/12)
**CCS, 2018**	Astra Zeneca (13/22)	Bayer (8/22)	BI (6/22)	Novartis (5/22)	Sanofi (5/22)
**CCS, 2018(AF)**	Bayer (18/25)	Pfizer (17/25)	BI (12/25)	Servier (10/25)	Abbott (6/25)
**ESC, 2020(AF)**	Medtronic (13/25）	Bayer (12/25)	BI (10/25)	Abbott (9/25)	Pfizer (7/25)
**ESC, 2020**	Astra Zeneca (11/26)	Abbott (10/26)	Bayer (9/26)	Medtronic (9/26）	BS (8/26)
**ESC, 2019**	Medtronic (10/25）	Abbott (8/25)	BS (7/25)	BI (6/25)	Astra Zeneca (6/25)
**ESC, 2017**	Astra Zeneca (9/18)	Abbott (6/18)	Bayer (5/18)	Medtronic (5/18）	Pfizer (5/18)
**NICE, 2013**	BS (3/15)	Menarini (1/15)	/	/	/
**NHFA/CSANZ,2016**	Astra Zeneca (14/29)	Pfizer (6/29)	Sanofi (6/29)	Abbott (4/29)	Novartis (3/29)
**NHFA/CSANZ,2018**	Medtronic (6/18）	Pfizer (6/18)	Bayer (5/18)	BS (4/18)	BMY (4/18)
**JCS, 2013**	BI (10/11)	Bayer (9/11)	DS (8/11)	OP (3/11)	BMY (2/11)
**TSC, 2016**	BI (3/27)	Bayer (3/27)	DS (3/27)	Astra Zeneca (2/27)	Pfizer (2/27)
**TSC, 2018**	/	/	/	/	/

BI=Boehringer-Ingelheim; BS=Boston Scientific; BMY=Bristol Myers Squibb; DS=Daiichi Sankyo; OP= Otsuka Pharmaceutical
