# Supplementary material for: Critical Appraisal of Guidelines for Antithrombotic Therapy in Atrial Fibrillation Post-Percutaneous Coronary Intervention
Source: Glob Heart. 2022 Feb 23;17(1):14. doi: 10.5334/gh.1104 (PMC8877875; doi:10.5334/gh.1104)
Supplement: Table S2. — AGREE II domain and overall assessment for eligible guidelines. [file gh-17-1-1104-s2.pdf]

table S2

Table S2 AGREE II domain and overall assessment for eligible guidelines

Guidelines	D1	D2	D3	D4	D5	D6:
AHA/ACC, 2019	88.89%	59.26%	83.33%	77.78%	59.72%	94.44%
AHA/ACC, 2014	85.19%	77.78%	81.25%	70.37%	59.72%	97.22%
ACCF/AHA, 2013	77.78%	61.11%	79.17%	74.07%	65.28%	97.22%
Chest,2018	81.48%	64.81%	85.42%	83.33%	59.72%	94.44%
CCS, 2018	90.74%	59.26%	70.83%	100.00%	65.28%	69.44%
CCS, 2018(AF)	94.44%	59.26%	61.11%	94.44%	65.28%	66.67%
ESC, 2020(AF)	81.48%	75.93%	72.22%	88.89%	68.06%	94.44%
ESC, 2020	75.93%	68.52%	79.17%	90.74%	63.89%	91.67%
ESC, 2019	79.63%	59.26%	67.36%	79.63%	62.50%	94.44%
ESC, 2017	83.33%	62.96%	63.19%	83.33%	65.28%	94.44%
NICE, 2013	100.00%	81.48%	95.83%	75.93%	72.22%	100.00%
NHFA/CSANZ,2016	90.74%	68.52%	60.42%	94.44%	48.61%	97.22%
NHFA/CSANZ,2018	90.74%	72.22%	63.89%	66.67%	48.61%	97.22%
JCS, 2013	79.63%	48.15%	55.56%	61.11%	36.11%	80.56%
TSC, 2016	64.81%	46.30%	43.06%	83.33%	40.28%	52.78%
TSC, 2018	81.48%	50.00%	50.00%	83.33%	47.22%	63.89%
Mean, % (Range)	0.84 (0.65,1.00)	0.63 (0.46,0.81)	0.69 (0.43,0.96)	0.82 (0.61,1.00)	0.58 (0.36,0.72)	0.87 (0.53,1.00)
ICC (95%CI)	0.86 (0.77–0.92)	0.94 (0.90–0.96)	0.94 (0.93–0.96)	0.91 (0.86–0.95)	0.95 (0.93–0.97)	0.96 (0.93–0.98)
